# Internal hernia in a patient with chylous ascites: a case report

**DOI:** 10.3389/fmed.2025.1640485

**Published:** 2025-11-24

**Authors:** Tao Wang, Lei Li, Yifei Wang

**Affiliations:** First Affiliated Hospital of Hebei North University, Zhangjiakou, China

**Keywords:** case report, chylous ascites, internal hernia, abdominal cavity, laparoscopic exploration

## Abstract

**Background:**

Chylous ascites is a rare medical disorder in which chylous fluid leaks from the lymphatic system into the abdominal cavity. When a patient has a history of abdominal surgery, especially subtotal gastrectomy, it is crucial for the physician to remain vigilant regarding the risk of internal hernia, which may result in lymphatic blockage and subsequent chylous ascites.

**Case presentation:**

A 70-year-old male admitted to our hospital complained of abdominal pain for 9 h. The patient had no obvious cause of abdominal pain, and the abdominal pain was not severe. The surgical history indicated a subtotal gastrectomy (Billroth II type) for a gastric ulcer that was conducted 20 years prior. CT scan revealed mild intestinal dilatation within the abdominal cavity and abdominal fluid accumulation. The diagnostic abdominal paracentesis was performed, revealing triglyceride levels that indicated the presence of chylous ascites. The clinicians opted for a conservative approach and executed percutaneous drainage. Two days later, the abdominal discomfort persisted, and around 600 mL of chylous ascites was being drained each day. Given that the prior conservative approach did not yield results, the subsequent course of action was laparoscopic exploration. An adhesive band was formed at the mesentery 1.5 meters from the Treitz ligament, accompanied by an internal hernia. The chylous fluid was drained, and the internal hernia was resected. The patient gradually resumed eating and was discharged after recovery.

**Conclusion:**

Chylous ascites is diagnosed through paracentesis and triglyceride testing. In patients with a prior abdominal surgery, particularly those who have undergone subtotal gastrectomy, it is prudent to consider the possibility of an internal hernia, even in the presence of negative imaging results. Conservative management frequently proves inadequate, while laparoscopic exploration serves a dual purpose of diagnosis and treatment, facilitating adhesiolysis and hernia reduction.

## Introduction

Chylous ascites is a rare clinical illness that can cause prolonged abdominal pain and is sometimes misdiagnosed with suppurative peritonitis (1). Neglecting the disease may result in delays in diagnosis and treatment. The most effective management strategy for idiopathic chylous ascites, whether it be conservative or surgical, is still not clearly defined. In cases where an internal hernia leads to chylous ascites, the most prudent course of action is surgical intervention. Internal hernia mainly involves the intestines, which can obstruct the digestive tract or even necrosis of the intestines (2). Internal hernia may lead to chyle leakage in the abdominal cavity (3). This document presents a case study of an internal hernia accompanied by chylous ascites. The article adheres to the CARE reporting guidelines (4).

### Case presentation

A 70-year-old male presented to our hospital complaining of abdominal pain for 9 h. He had a history of coronary heart disease and had undergone a subtotal gastrectomy (Billroth II type) for gastric ulcer 20 years ago. The patient presented with sudden onset of persistent abdominal pain without an apparent etiology. There were no accompanying symptoms such as fever, nausea, or vomiting, leading to the decision to admit the patient to our emergency department. Upon initial assessment, the patient exhibited alertness and consciousness, with a recorded body temperature of 36.5 °C, blood pressure measuring 130/70 mmHg, a heart rate of 80 beats per minute, a respiratory rate of 22 breaths per minute, and a blood oxygen saturation level of 95% in ambient air. Cardiac and thoracic percussion and auscultation showed no abnormality. The patient demonstrated standard developmental milestones, experienced moderate malnutrition, displayed a troubled demeanor, and was conveyed on a level surface. There was no icterus, pallor, lymphadenopathy, cyanosis, clubbing, edema, and the patient was well hydrated. The abdomen was slightly distended, without abdominal varicose vein, gastrointestinal type, and peristaltic wave. There were slight distention of the whole abdomen, mild tenderness, no rebound pain, and no abdominal muscle tension. The examination revealed no enlargement of the liver, gallbladder, or spleen, and no masses were detected in the groin region. Abdominal percussion yielded a drum-like sound, with no signs of mobile dullness. Auscultation of bowel sounds was weak with no hyperactivity. The digital rectal examination did not touch the mass, and the withdrawal of the finger sheath was not stained with blood. The laboratory findings indicated minor reductions in hemoglobin (106 g/L) and albumin (30.8 g/L) concentrations, while all other assessments remained within normal limits.

**Figure 1:**
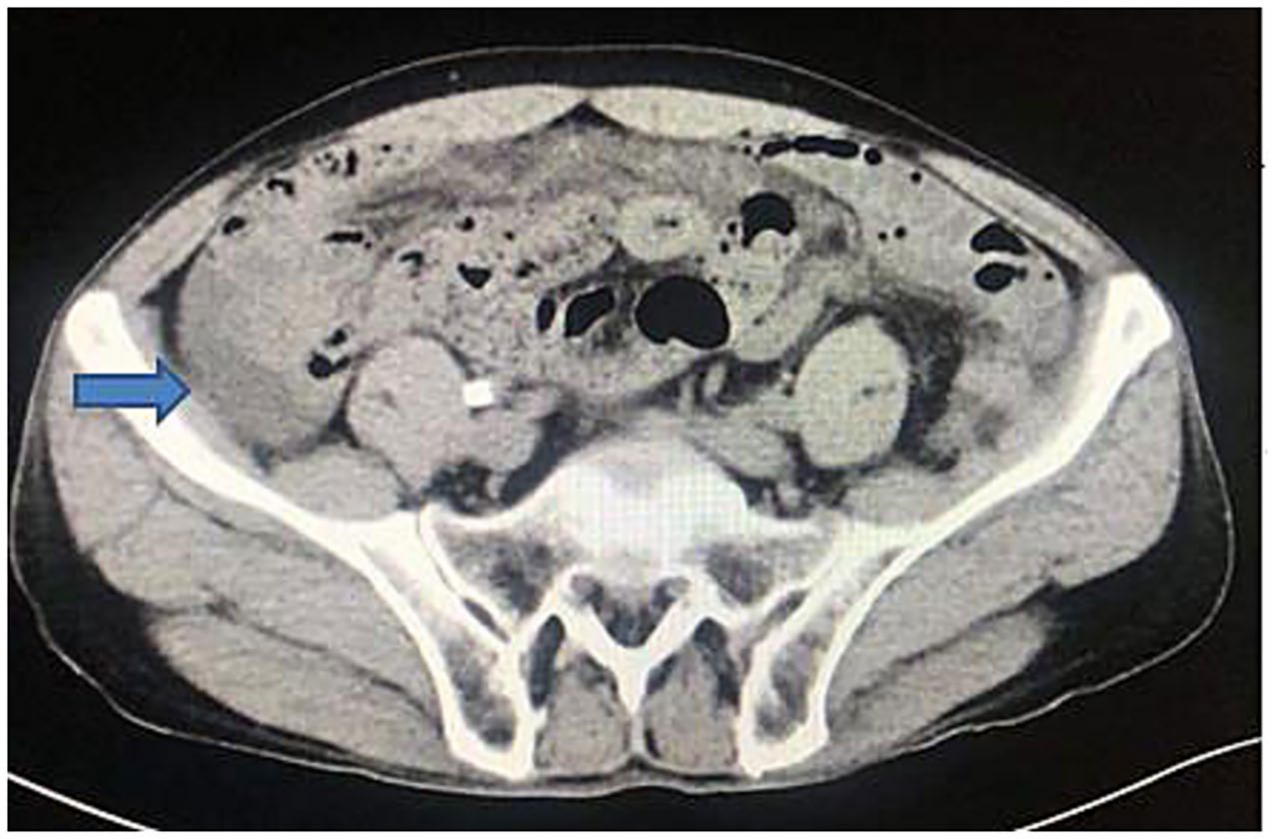
Abdominal computed tomography showing abdominal fluid.

**Figure 2:**
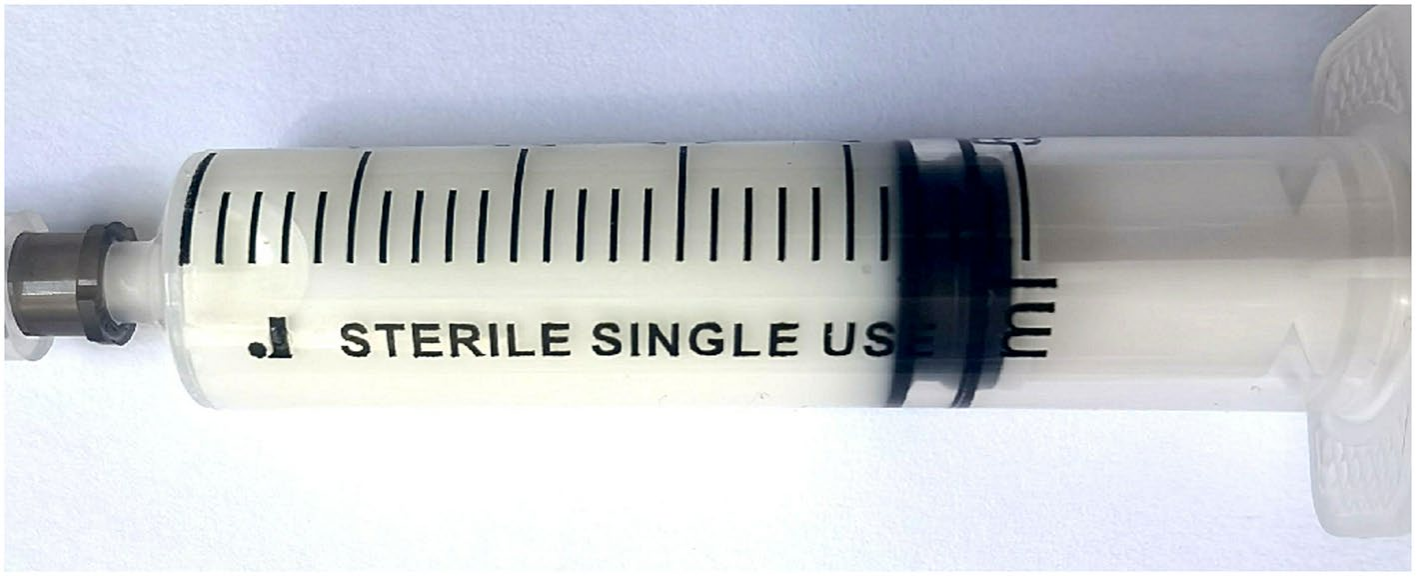
Opalescent ascites from abdominal paracentesis.

Abdominal computed tomography (CT) revealed mild intestinal dilatation within the abdominal cavity and abdominal fluid accumulation (Figure 1). Abdominal CT showed no tumor and cirrhosis. Abdominal color ultrasound showed no mesenteric vein embolism. A diagnostic abdominal paracentesis was performed, and opalescent ascites were visible (Figure 2). Subsequently, a percutaneous drainage procedure was conducted, resulting in the extraction of 400 mL of chylous fluid. Relevant laboratory tests examined the chylous fluid. The specific gravity of chylous fluid was 1.018, pH was alkaline (pH = 7.62), and protein content was 35 g/L. The cell count was low, with mostly lymphocytes (4.2 × 10^9^/L) and a few neutrophils (0.8 × 10^9^/L). The bacterial culture was negative. Droplets of fat could be seen under the microscope. Chylous fluid had high triglyceride content (3.85 g/L) and low cholesterol content (0.74 g/L), and cholesterol/triglyceride <1.0, leading to the diagnosis of chylous ascites. The patient did not present with any other significant illnesses at the time, prompting the implementation of conservative regimen measures such as water fasting, cefoperazone, somatostatin, and nutritional liquid containing glucose, fat emulsion, amino acids, vitamins, and trace elements. However, the abdominal pain was not relieved after 2 days, with about 600 mL of chylous ascites drained daily. Following the ineffectiveness of previous conservative regimen, laparoscopic exploration was considered the subsequent course of action.

**Figure 3:**
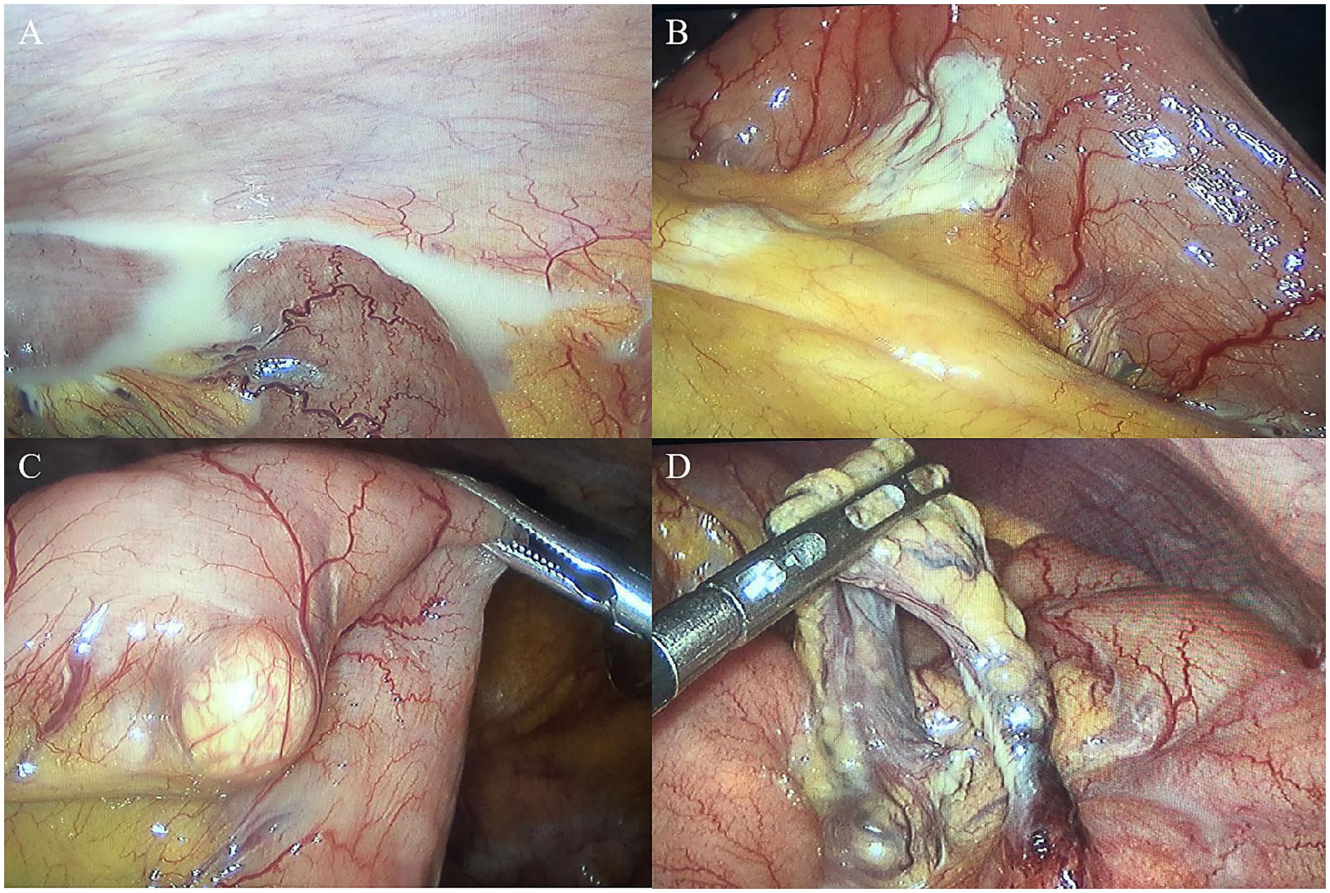
Laparoscopic aspect **(A)**. Chylous ascites can be seen in the abdominal cavity **(B)**. Scattered white patches in the mesentery **(C)**. White bulges in the mesentery **(D)**. An adhesive band with internal hernia.

**Figure 4:**
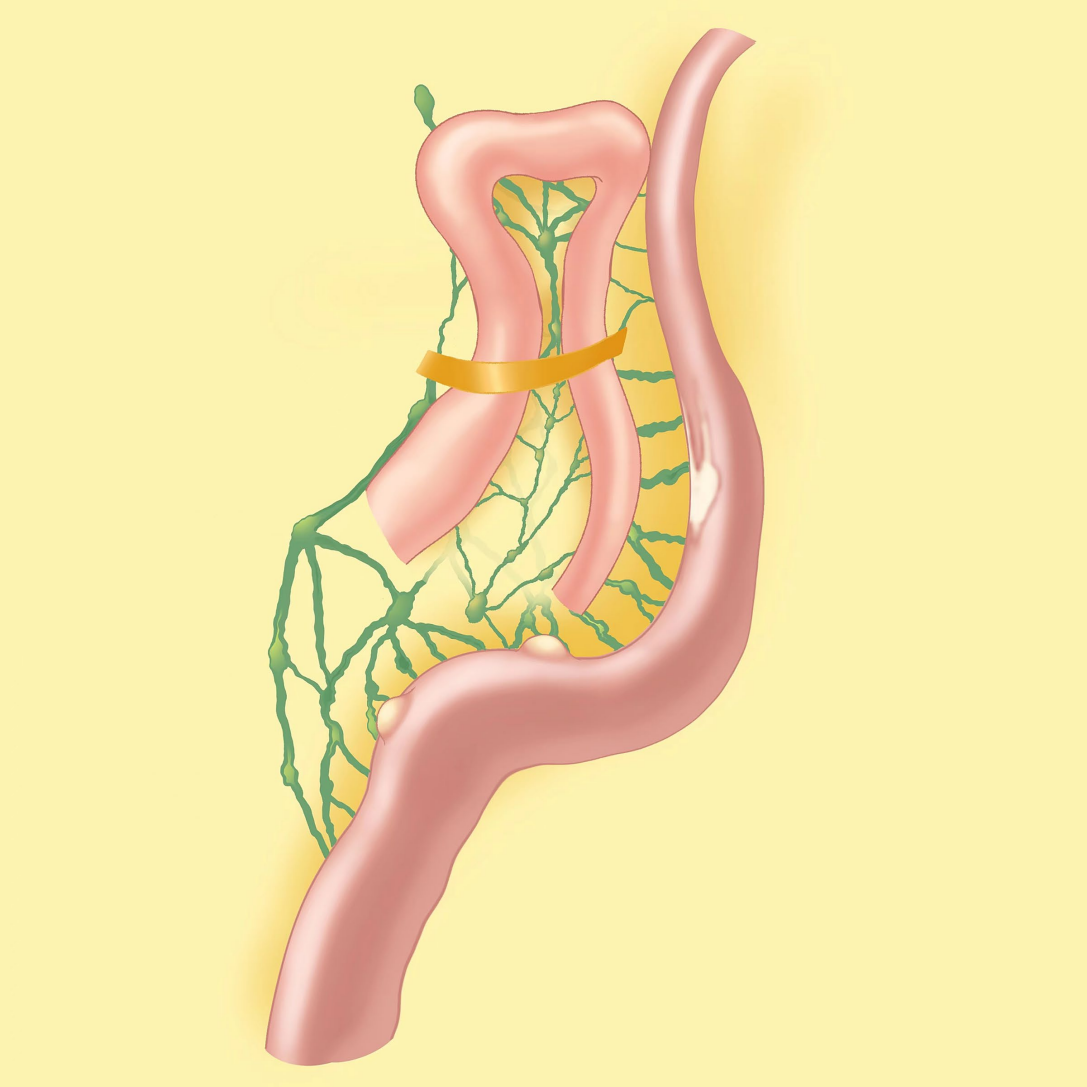
A simplified anatomical schematic.

We selected the navel as the observation aperture and the outer boundary of the rectus muscle in the right upper and lower abdomen as the surgical entry point. Through laparoscopic exploration, we found approximately 150 mL of chylous ascites (Figure 3A) in the abdominal cavity, in addition to scattered white patches (Figure 3B) and white bulges (Figure 3C) in the mesentery, but no rupture was found. No perforation sites were identified in the stomach, small intestine, or colon. Both ends of the adhesive band (Figure 3D) were fixed at the mesentery about 1.5 m from the Treitz ligament, and an internal hernia formed. The small intestine, with a length of approximately 20 cm, exhibited herniation adjacent to the adhesive band, while the proximal segment of the small intestine displayed slight dilation. A simplified anatomical schematic was added to illustrate the hernia location and its relationship to lymphatic drainage (Figure 4). All the chylous fluid present within the abdominal cavity was evacuated. The adhesive band was meticulously severed at both ends along the root using a Johnson ultrasonic knife, subsequently extracted from the body via laparoscopic trocar. No intestinal necrosis was present, so enterectomy was not performed. The abdominal cavity was irrigated with warm saline until clarification; no active bleeding occurred, and no chylous fluid continued to effuse. A rubber tube was placed in the pelvic cavity for drainage and was extracted from the right abdominal wall. The chylous fluid was excised, the internal hernia was addressed, and the surgery completed. The patient underwent postoperative water fasting and received cefoperazone, somatostatin and intravenous nutrition treatment containing glucose, fat emulsion, amino acids, vitamins, and trace elements. On the first day following surgery, 50 mL of light red chylous fluid was drained from the abdomen through the drainage tube; on the second day, 30 mL of light yellow fluid was discharged. Relevant laboratory tests examined the ascites on the second day. The specific gravity of chylous fluid was 1.017, pH was weakly alkaline (pH = 7.42), and protein content was 25 g/L. The number of cells was small, containing most white blood cells (60 × 10^6^/L). The bacterial culture was negative. Droplets of fat could barely be seen under the microscope. The ascites had low triglyceride content (0.32 mmol/L). On the third day, 10 mL of yellow and transparent fluid was drained; on the fourth day, there was no obvious effusion in the abdominal color ultrasound re-examination, and the drainage tube was removed. The patient gradually resumed eating and recovered after surgery. He was discharged on the 7th day after surgery. After 6 months of follow-up, the patient did not complain of any discomfort. The re-examination of abdominal CT showed no obvious abnormalities. A figure showcasing a timeline with relevant data from episode of care is attached (Supplementary Figure 1).

## Discussion

Chylous ascites is a rare disorder characterized by ascites rich in chylomicrons in the abdominal cavity. Chylous ascites is caused by the compression, obstruction, or rupture of thoracic ducts, lymphatic vessels and its branches, causing chylous fluid to enter the abdominal cavity (5). In developed countries, two-thirds of chylous ascites are caused by abdominal malignancies and cirrhosis of the liver, while infectious diseases account for the majority in developing countries (5). Chylous ascites caused by internal hernia are rare. An internal hernia occurs when abdominal organs are displaced, often resulting in the obstruction of small bowel loops as they move through a peritoneal or mesenteric opening into a restricted area within the abdominal cavity (2). Internal hernia is relatively rare in clinical practice and difficult to diagnose without specific symptoms. Internal hernia can cause gastrointestinal obstruction and even life-threatening intestinal necrosis.

Lymph is rich in proteins, fats, and lymphocytes; therefore, chylous ascites can result in significant losses of these components, leading to hypoalbuminemia, hypolipidemia, and a decrease in lymphocyte levels (6, 7). The diagnosis of chylous ascites ought to be complemented by ascitic fluid puncture and subsequent laboratory examination. Misdiagnosis and missed diagnosis of chylous ascites occur frequently in clinical practice. Their occurrence is frequently attributed to the absence of standard characteristics of ascites during abdominal examination and the unavailability of specific diagnostic tests for ascites in certain medical facilities. However, it should be noted that it is necessary to distinguish between chylous ascites and other ascites. Some ascites are similar to chylous ascites. Still, they slowly formed serous ascites in chronic abdominal infection. The fatty degeneration of the pus cells present in the ascites results in a chylous appearance, with sediment accumulating at the bottom after a period of repose. Its chemical components are lecithin, cholesterol, and cytoclasts. Diagnosis of this condition can be confirmed through quantitative triglyceride determination and microscopic examination of fluid stained with Sudan III (8). Furthermore, it is important to note that not every instance of chylous ascites consists of chyle. Some manifestations may appear as light yellow or thin fluid, attributed to low triglyceride levels (9). Currently, there are no guidelines to elucidate the diagnostic criteria and treatment of chylous ascites. The diagnosis relies on the manifestation of ascites and the concentration of triglycerides. Most studies adopted the level of triglyceride exceeding 2.26 mmol/L (200 mg/dL) as the diagnostic criteria. Regardless of whether the ascites appear as light yellow or thin fluid, an elevation in triglyceride levels beyond 2.26 mmol/L (200 mg/dL) categorizes the ascites as chylous. Radionuclide imaging and X-ray lymphography are established clinical techniques for diagnosing chylous ascites. These methods offer a clearer visualization of lymphatic vessels and the locations of any lymphatic leakage or obstruction, thereby providing a solid foundation for planning surgical interventions (10, 11).

Acute chylous ascites enter the abdominal cavity quickly, causing chemical peritonitis, a condition that is easily mistaken as a variety of other acute abdominal ailments, such as gastrointestinal perforation and acute appendicitis (12). Most chylous ascites are treated conservatively in their early stage. Given that the majority of adult chylous ascites cases are secondary, it is essential to actively address the underlying primary disease. Conservative treatment should focus on the primary condition while also restricting the patient’s salt and water consumption to promote diuresis and correct hypoproteinemia. The patients were given low fat, low sodium, high protein diet, and even total parenteral nutrition support combined with somatostatin (5). Ascitic fluid can be effectively managed through puncture and drainage when significant abdominal fluid accumulation leads to compression symptoms. Surgical treatment comprises laparoscopic exploration with repair and closure of the lymphatic rupture or removal of lymphatic obstruction factors, or percutaneous embolization employing embolization agents in combination with lymphangiography (5).

In this case, CT and color ultrasound examination revealed the presence of ascites in the abdomen. Nevertheless, no definitive cause was identified, and the potential for tumor, cirrhosis, and mesenteric vein embolism resulting in ascites was also excluded. The physical examination revealed an absence of mobile dullness, suggesting that the volume of ascites was minimal. The physical examination failed to reveal any signs of peritonitis, thereby excluding the likelihood of an abdominal abscess or perforation. Opalescent ascites were discovered during the abdominal puncture, and the test revealed that the triglyceride levels were high, indicating that the ascites were chylous. The amount of chylous ascites did not decrease after conservative treatment, and laparoscopic exploration was the next treatment option. It was found that an internal hernia had developed due to the formation of an adhesive band after abdominal surgery. The internal hernia was not severe and did not result in intestinal necrosis. The patient showed signs of recovery after the surgical removal of the abdominal adhesion band, indicating that the root cause of the abdominal chylous leakage was the internal hernia. Abdominal adhesion bands have the potential to obstruct abdominal lymphatic reflux, leading to the overflow of chylous fluid into the abdominal cavity and the subsequent formation of chylous ascites (13). Prompt interventions are crucial for alleviating adhesions and addressing lymphatic vessel blockages to facilitate recirculation. When chylous ascites are suspected, relevant laboratory tests, including detecting triglyceride content in ascites, are feasible to make a definite diagnosis. In the absence of appropriate detection methods at the hospital, the assessment can solely rely on the color and characteristics of the ascites. When chylous ascites are diagnosed, the choice of conservative or surgical treatment depends on whether the cause of chylous ascites can be relieved by surgical treatment. In cases where abdominal tumor compression of lymphatic vessels is the underlying issue, tumor removal is necessary to alleviate lymphatic pressure and effectively address chylous ascites. In the event that an abdominal infection leads to chylous ascites, it is imperative to address the condition through anti-infective therapy or surgical intervention to eliminate the source of infection. If cirrhosis causes chylous ascites, we need to treat it with symptomatic measures such as liver protection and correction of hypoproteinemia. If it can be confirmed that chylous ascites are caused by internal hernia through relevant tests, we will consider surgery first. When we cannot find the cause of chylous ascites, conservative treatment is considered first. In a patient with a history of abdominal surgery, especially subtotal gastrectomy, clinicians should be highly vigilant about the possibility of adhesive bands causing an internal hernia, although CT does not detect an internal hernia. Should conservative treatment be selected at this juncture, the resolution of chylous ascites would prove challenging, as the underlying etiology remains unaddressed. At this time, it can generally be treated by surgical removal of the etiology, placement of an abdominal drainage tube, and corresponding postoperative conservative treatment.

The mesentery, a fold of peritoneum, houses an extensive lymphatic network: initial lymphatic capillaries in intestinal villi absorb chyle (lipid-rich lymph), merging into larger mesenteric lymphatic vessels that run alongside mesenteric arteries/veins, draining into cisterna chyli. Internal hernia occurs when bowel loops herniate through congenital/acquired peritoneal defects. As herniated bowel and mesentery become entrapped, increased intra-abdominal pressure and mechanical compression narrow or occlude mesenteric lymphatic vessels. This obstruction disrupts chyle flow: proximal lymphatic dilation leads to vessel wall damage, and sustained pressure causes chyle extravasation into the peritoneal cavity, forming chylous ascites. Additionally, impaired lymphatic drainage reduces tissue fluid clearance, exacerbating mesenteric edema, which further compresses lymphatics—creating a vicious cycle that worsens lymphatic obstruction and ascites accumulation.

A search of English literature concerning case reports of chylous ascites with internal hernia reveals 8 cases (Table 1) (3, 14–19). Among these cases, there were 5 males and 3 females, with ages spanning from 29 to 85 years (mean age 44.1 ± 19.7 years). Seven of the 8 cases have surgical history. The diseases, operative procedures, symptom, abdominal CT and hernia site are presented in Table 1. The surgical interventions executed comprised five instances of Roux-en-Y gastric bypass, a singular case of anastomosis gastric bypass-minigastric bypass, and one instance of Billroth 2 gastrointestinal bypass surgery. There are three cases of whirl sign in abdominal CT, and four cases of Petersen’s space in hernia site. There is no intestinal necrosis and bowel resection in all eight cases.

**Table 1:** Case reports of chylous ascites associated with internal hernia.

No.	Author	Year	Age	Sex	Surgical history (disease, procedure)	Symptom	Abdominal CT	Hernia site	Bowel resection	OL/LL	Days^a^
1	Koyama (14)	2019	69	M	EGJ cancer, total gastrectomy with lower esophageal resection, LRGYB and cholecystectomy.	Dysphagia and abdominal pain	Local narrowing of the jejunal branch of SMA, SMV was completely blocked, whirl sign	Jejunojejunostomy mesenteric defect	No	LL	13
2	Abu (15)	2020	29	M	Bariatric surgery, OAGB MGB	Diffuse tenderness	Enlargement of lymph nodes at the mesentery	Petersen’s space	No	LL	4
3	Akama (16)	2016	85	M	Gastric cancer, distal gastrectomy, LRGYB	Focal tenderness and distention in the epigastrium	Dilation of the transverse colon, severe ascites, whirl sign	Petersen’s space	No	OL	16
4	Sami (17)	2023	34	M	Bariatric surgery, LRGYB	Severe abdominal pain with radiation to the right flank	Whirl sign	Petersen’s space	No	LL	ND
5	Yu (18)	2015	40	F	ND	Severe, colicky abdominal pain	A cluster of dilated proximal small bowel loops with ischemic change	Left paraduodenal hernia	No	OL	6
6	Zaidan (3)	2018	29	M	Bariatric surgery, LRGYB	Sharp, severe abdominal pain in RUQ	ND	Mesenteric defect between alimentary limb and biliopancreatic limbs	No	LL→OL	4
7	Azorín (19)	2021	31	F	Bariatric surgery, B-II	Abdominal pain of duration	ND	Petersen’s space	No	LL	3
8	Azorín (19)	2021	36	F	Bariatric surgery, LRGYB	Continuous abdominal pain, nausea and vomiting.	Transmesenteric internal hernia	Mesenteric gap of the loop foot	No	LL→OL	6

M, male; F, female; EGJ, esophagogastric junction; OAGB-MGB, One anastomosis gastric bypass-minigastric bypass; LRGYB, Roux-en-Y gastric bypass; B-II Billroth 2, gastrointestinal bypass surgery; RUQ, right upper quadrant; SMA, superior mesenteric artery; SMV, superior mesenteric vein; OL, open laparotomy; LL, laparoscopic laparotomy; ND, not described; Days^a^ number of days from surgery to hospital.

Our 70-year-old male case aligns with the eight reported internal hernia-related chylous ascites cases in core features: abdominal pain as the primary symptom, prior abdominal surgery (Billroth II subtotal gastrectomy vs. five cases of LRGYB, one case of OAGB-MGB, one case of B-II in reports), and no need for bowel resection. However, key differences emerge: (1) MOST reported cases (5/8) had bariatric surgery, while ours involved gastric ulcer surgery—highlighting internal hernia risk in non-bariatric abdominal procedures. (2) Our CT showed only mild bowel dilatation and ascites, and diagnosis relied on abdominal paracentesis and triglyceride detection, unlike five reports with definitive CT signs. (3) Conservative treatment failed in our case, while other cases did not receive conservative treatment.

In cases where patients present with chylous ascites and possess a background of abdominal surgery, particularly subtotal gastrectomy, it is plausible that laparoscopic exploration represents the most effective therapeutic approach. In addition, if the patient’s chylous ascites are not healed, we need percutaneous embolization using embolization agents in combination with lymphangiography. In the case of a patient with a history of Roux-en-Y gastric bypass procedure (RYGBP), the occurrence of chylous ascites indicates that the small bowel obstruction is probably connected to an internal hernia via a patent Petersen’s defect (17, 20). In patients undergoing gastric surgery with chylous ascites, internal hernia caused by adhesion band is less common than Petersen’s hernia. Chylous ascites may be a reliable sign of intestinal viability for herniated intestines (14), and the small intestine was not necrotic in our study.

This study has several limitations. First, as a single-case report, its findings lack generalizability. Further multi-case studies are warranted to validate these observations. Second, the diagnostic process relies heavily on clinical judgment in the absence of definitive preoperative imaging evidence for internal hernia.

## Conclusion

In instances where patients present with abdominal pain alongside CT-verified ascites, it is imperative to perform diagnostic paracentesis and evaluate triglyceride levels to determine the existence of chylous ascites. In the absence of a definitive cause, it is prudent to continue with the previously established conservative regimen. However, even without CT evidence, persons with a history of abdominal surgery, particularly subtotal gastrectomy, should be cautious of an internal hernia caused by an adhesive band. In many instances, conservative treatment proves inadequate, necessitating timely laparoscopic exploration, which serves both diagnostic and therapeutic purposes, facilitating adhesiolysis and hernia reduction.

## Data Availability

The datasets presented in this study can be found in online repositories. The names of the repository/repositories and accession number(s) can be found in the article/Supplementary material.

## Supplementary material

The Supplementary material for this article can be found online at: https://www.frontiersin.org/articles/10.3389/fmed.2025.1640485/full#supplementary-material

SUPPLEMENTARY FIGURE 1 A timeline with relevant data from episode of care.
