# Supplementary figures and images for: Internal hernia in a patient with chylous ascites: a case report
Source: Front Med (Lausanne). 2025 Nov 24;12:1640485. doi: 10.3389/fmed.2025.1640485 (PMC12682740; doi:10.3389/fmed.2025.1640485)

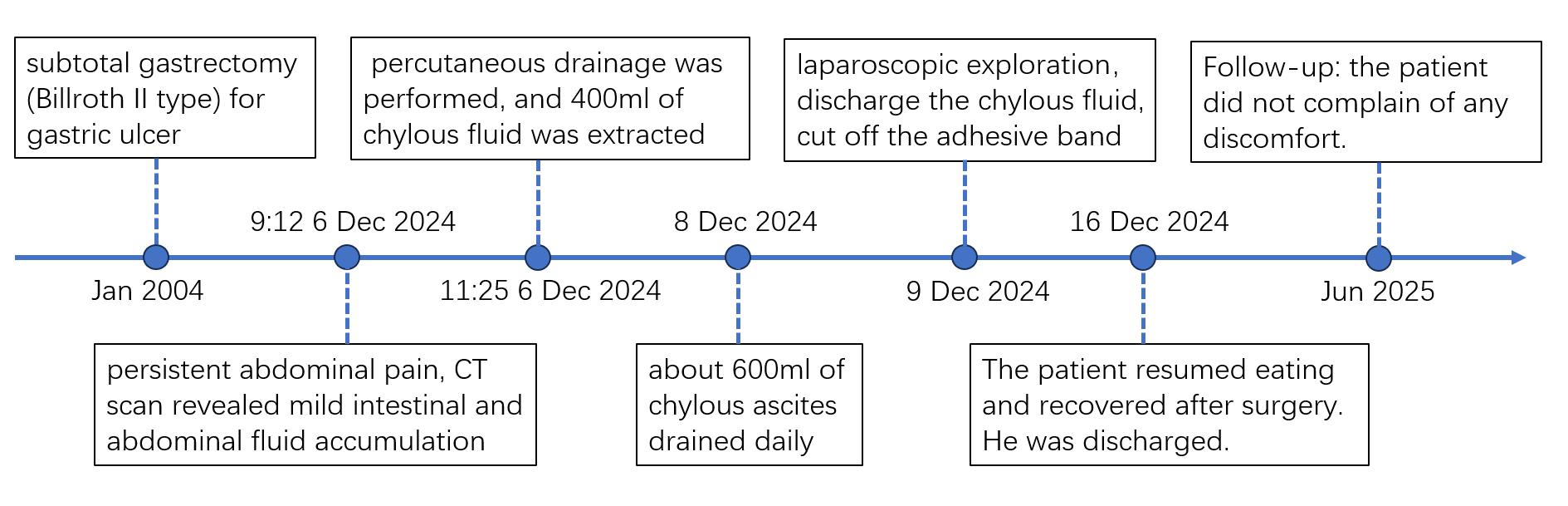

Supplement: SUPPLEMENTARY FIGURE 1 — A timeline with relevant data from episode of care. [file Image_1.JPEG]
